# Palmitoylethanolamide counteracts substance P-induced mast cell activation in vitro by stimulating diacylglycerol lipase activity

**DOI:** 10.1186/s12974-019-1671-5

**Published:** 2019-12-26

**Authors:** Stefania Petrosino, Aniello Schiano Moriello, Roberta Verde, Marco Allarà, Roberta Imperatore, Alessia Ligresti, Ali Mokhtar Mahmoud, Alessio Filippo Peritore, Fabio Arturo Iannotti, Vincenzo Di Marzo

**Affiliations:** 1 Endocannabinoid Research Group, Istituto di Chimica Biomolecolare, Consiglio Nazionale delle Ricerche, Via Campi Flegrei 34, 80078 Pozzuoli (Napoli), Italy; 2 Epitech Group SpA, Via Einaudi 13, 35030, Saccolongo (Padova), Italy; 3 Canada Excellence Research Chair on the Microbiome-Endocannabinoidome Axis in Metabolic Health, CRIUCPQ and INAF, Faculties of Medicine and Agriculture and Food Sciences, Université Laval, Quebéc City, Canada

**Keywords:** 2-arachidonoylglycerol, Diacylglycerol lipase, Mast cells, Neuroinflammation, Palmitoylethanolamide

## Abstract

**Background:**

Palmitoylethanolamide (PEA) is a pleiotropic endogenous lipid mediator currently used as a “dietary food for special medical purposes” against neuropathic pain and neuro-inflammatory conditions. Several mechanisms underlie PEA actions, among which the “entourage” effect, consisting of PEA potentiation of endocannabinoid signaling at either cannabinoid receptors or transient receptor potential vanilloid type-1 (TRPV1) channels. Here, we report novel molecular mechanisms through which PEA controls mast cell degranulation and substance P (SP)-induced histamine release in rat basophilic leukemia (RBL-2H3) cells, a mast cell model.

**Methods:**

RBL-2H3 cells stimulated with SP were treated with PEA in the presence and absence of a cannabinoid type-2 (CB2) receptor antagonist (AM630), or a diacylglycerol lipase (DAGL) enzyme inhibitor (OMDM188) to inhibit the biosynthesis of the endocannabinoid 2-arachidonoylglycerol (2-AG). The release of histamine was measured by ELISA and β-hexosaminidase release and toluidine blue staining were used as indices of degranulation. 2-AG levels were measured by LC-MS. The mRNA expression of proposed PEA targets (*Cnr1, Cnr2*, *Trpv1*, *Ppara* and *Gpr55*), and of PEA and endocannabinoid biosynthetic (*Napepld, Dagla* and *Daglb*) and catabolic (*Faah*, *Naaa* and *Mgl*) enzymes were also measured. The effects of PEA on the activity of DAGL-α or -β enzymes were assessed in COS-7 cells overexpressing the human recombinant enzyme or in RBL-2H3 cells, respectively.

**Results:**

SP increased the number of degranulated RBL-2H3 cells and triggered the release of histamine. PEA counteracted these effects in a manner antagonized by AM630. PEA concomitantly increased the levels of 2-AG in SP-stimulated RBL-2H3 cells, and this effect was reversed by OMDM188. PEA significantly stimulated DAGL-α and -β activity and, consequently, 2-AG biosynthesis in cell-free systems. Co-treatment with PEA and 2-AG at *per se* ineffective concentrations downmodulated SP-induced release of histamine and degranulation, and this effect was reversed by OMDM188.

**Conclusions:**

Activation of CB2 underlies the inhibitory effects on SP-induced RBL-2H3 cell degranulation by PEA alone. We demonstrate for the first time that the effects in RBL-2H3 cells of PEA are due to the stimulation of 2-AG biosynthesis by DAGLs.

## Background

Palmitoylethanolamide (PEA) was initially identified from the purified lipid fractions of egg yolk [1], and later found in a wide variety of food sources [2, 3]. In addition, PEA is also considered an endogenous lipid mediator produced *on demand* in several mammalian cell types and tissues to counteract inflammatory and other noxious responses [2]. Accordingly, PEA tissue concentrations are altered during several inflammatory disorders [2, 4]. For example, an increase of PEA levels was found both in human HaCaT keratinocytes stimulated with polyinosinic polycytidylic-acid (poly-(I:C)), an in vitro model of allergic contact dermatitis (ACD), and in the ear skin of 2,4-dinitrofluorobenzene (DNFB)-sensitized and challenged mice, an *in vivo* model of the early phase of ACD characterized by activation of keratinocytes [5]. Increased PEA levels were also found in the skin of dogs with atopic dermatitis [6]. On the other hand, decreased PEA levels were reported in granuloma in rats, a model of chronic inflammation sustained by neoangiogenesis [7], and in spinal and supraspinal brain regions involved in nociception in mice with neuropathic pain [8]. Therefore, while the increase of endogenous PEA levels in some disorders might be a compensatory response aiming at counteracting inflammatory processes, their decrease in other pathological conditions could contribute to the etiology of the disease.

In agreement with this hypothesis, exogenously administered PEA in the micrometer particle size range potentiates endogenous anti-inflammatory mechanisms in experimental models as well as in the clinic [2, 4, 9, 10]. In granuloma, PEA reduced inflammatory hallmarks, including tumor necrosis factor (TNF)-α and granuloma-dependent angiogenesis [7]. Likewise, PEA inhibited the expression and release of the pro-inflammatory chemokine monocyte chemotactic protein-2 (MCP-2) in poly-(I:C)-stimulated HaCaT cells in vitro, as well as DNFB-induced ear inflammation in mice during the early and late phase of ACD, the latter being characterized by activation of mast cells (MC) [5, 11]. The anti-inflammatory effects of PEA in the early and late phase of ACD were blocked by antagonism at the transient receptor potential vanilloid type-1 (TRPV1) channels and cannabinoid receptor type-2 (CB2), respectively, despite the fact that the compound is inactive per se at both these targets [12, 13]. Therefore, these effects were explained with the capability of PEA to elevate the levels or actions of endogenous agonists at cannabinoid receptors and TRPV1 receptors, i.e., anandamide (AEA) and oleoylethanolamide (OEA) [5, 14–16], and hence to exert an indirect receptor-mediated mechanism, known as the *entourage effect* [13, 17, 18]. Accordingly, PEA had been previously shown to increase either the endogenous levels [19], or the actions at TRPV1 channels [13, 18], of AEA, and, more recently, to enhance the endogenous levels of, and the activation/desensitization of TRPV1 by, 2-arachidonoylglycerol (2-AG) [20], another endogenous lipid capable of activating both cannabinoid receptors and TRPV1 [21]. A stimulatory effect on 2-AG levels was recently suggested to occur also in the brain, following direct activation of G protein-coupled receptor 55 (GPR55) by PEA [22]. PEA was recently found to also elevate CB2 expression in microglia through direct activation of the peroxisome proliferator-activated nuclear receptor-α (PPARα) [23], a well-established direct target of the lipid [10, 24]. Indeed, the aforementioned stimulatory effect of PEA on AEA activation of TRPV1 was later shown to be due to the activation of PPARα and subsequent sensitization by the latter of TRPV1 [25, 26]. In summary, several direct or indirect receptor- and endocannabinoid/endovanilloid-mediated mechanisms, often in sequence or synergy with each other, have been proposed to explain the many CB2- and TRPV1-dependent effects of PEA [2].

Historically, the first, and possibly most important, anti-inflammatory effect of PEA to be ascribed to CB2 activation was the downregulation of MC degranulation, which was described in a widely used MC model, the rat basophilic leukemia (RBL-2H3) cells [27], when evidence for the lack of direct effect of the lipid on cannabinoid receptors was not yet available. Indeed, the negative control of MC activity is one of the most commonly suggested cellular mechanisms for the protective actions of PEA in vivo, among which the above mentioned inhibitory effects on granuloma and late phase ACD [7, 11], and its counteraction of neurogenic inflammation (NI) and inflammatory and neuropathic pain [28–32]. Nevertheless, the exact mechanism through which PEA modulates MC degranulation is still unknown. Is this effect due to the upregulation of CB2 expression, as recently found in microglia [23]? Or is it due to elevation of the levels or activity of endocannabinoids, and in particular 2-AG, as shown in keratinocytes and brain neurons [20, 22], given the much higher efficacy of this compound, compared to AEA, at CB2 receptors [12, 33]?

In order to provide an answer to these questions, we investigated the mechanism(s) through which PEA counteracts substance P (SP)-induced RBL-2H3 cell degranulation, and, in particular, the possibility that it does so by enhancing 2-AG levels. It is well known that 2-AG is mostly biosynthesized by two diacylglycerol lipases (DAGL)-α and - β[4], and degraded to arachidonic acid and glycerol by monoacylglycerol lipase (MGL) [34]. Therefore, along with other possible molecular effects of PEA, we have assessed for the first time in different in vitro settings its possible stimulatory or inhibitory effects, respectively, on these enzymes, and the consequences of DAGL stimulatory effects on 2-AG biosynthesis by PEA in RBL-2H3 cells.

## Methods

### Materials and reagents

All reagents were purchased from Sigma-Aldrich (Milano, Italy) unless otherwise specified. RBL-2H3 cell line was purchased from LGC Standards (Milano, Italy). PEA in an ultra-micronized formulation was provided by the Epitech Group SpA (Saccolongo, Padova, Italy). PEA, when inserted in water after being dissolved in methanol, remained water-soluble up to 25 μM. AM630 and JWH133 were purchased from Tocris Bioscience (Space Import-Export, Milano, Italy). 2-AG was purchased from ENZO Life Sciences (Roma, Italy). OMDM188 was a kind gift from Dr. Giorgio Ortar (Sapienza Università di Roma, Roma, Italy). Deuterated standards-^2^[H]_8_-AEA, [^2^H]_5_-2-AG and [^2^H]_4_-PEA-were purchased from Cayman Chemical (Cabru, Arcore, Italy). Histamine ELISA Kit was purchased from Abnova (Prodotti Gianni, Milano, Italy). Cyclic AMP assay was purchased from Eurofins-DiscoverX (Fremont, CA). MultiTox-Glo Multiplex Cytotoxicity kit was purchased from Promega Corporation (Promega Italia, Milano, Italy).

### Cell cultures

RBL-2H3 cells were grown in Eagles Modified Essential Medium (EMEM) supplemented with glutamine (2 mM), penicillin (50 U/ml), streptomycin (50 μg/ml) and 15% fetal bovine serum (FBS), in a humidified 5% CO_2_ atmosphere at 37 °C, plated on 100 mm diameter Petri dishes.

### SP-induced NI in RBL-2H3 cells

RBL-2H3 cells were plated into 24-well culture dishes at a cell density of 2 × 10^5^ cells per well, or into 6-well culture dishes at a cell density of 9 × 10^5^ cells per well, for 1 day at 37 °C in 5% CO_2_ atmosphere. After 1 day, RBL-2H3 cells were stimulated with SP (10 μM) or vehicle (water) and incubated for 15 min at 37 °C in 5% CO_2_ atmosphere.

### β-Hexosaminidase release assay

SP-stimulated RBL-2H3 cells (2 × 10^5^ cells/well) were treated with PEA (0.1, 0.5, 1, and 10 μM) or vehicle (methanol, max 0.1%) for 15 min at 37 °C in 5% CO_2_ atmosphere. After 15 min, the supernatants (15 μl) were transferred to 96-well plates and incubated with 60 μl of substrate (1 mM *p*-nitrophenyl-*N*-acetyl-β-D-glucosaminide in citrate 0.05 M, pH 4.5) for 1 h at 37 °C. To determine the total amount of released β-hexosaminidase, the cells were lysed with 0.1% Triton X-100 and incubated with substrate using the same procedure as for the determination of the activity in the supernatants. The reaction was stopped by adding 150 μl of 0.1 M sodium bicarbonate buffer (pH 10.0), and the reaction product was monitored by measuring the optical density (OD) at 405 nm by using a reader GENios Pro (Tecan). The results were expressed as % of the total β-hexosaminidase content of the cells determined by cell lysis with 0.1% Triton X-100, and calculated by using the following formula: % degranulation = [OD_supernatant_/(OD_supernatant_ + OD_triton *x*−100_)] × 100.

### Histamine release assay

SP-stimulated RBL-2H3 cells (2 × 10^5^ cells/well) were treated with PEA (10 μM) or vehicle (methanol) for 15 min at 37 °C in 5% CO_2_ atmosphere. SP-stimulated RBL-2H3 cells were also treated with a CB2 antagonist, AM630 (0.1 μM), in the presence and absence of PEA (10 μM), or JWH133 (0.1 μM) (a CB2 synthetic agonist), and incubated for the indicated time. SP-stimulated RBL-2H3 cells were also treated with 2-AG (0.1 and 1 μM), or co-treated with PEA (0.1 μM) and 2-AG (0.1 μM), and incubated for the indicated time. SP-stimulated RBL-2H3 cells were also co-treated with PEA (10 μM) and OMDM188 (10 μM) (a DAGL inhibitor), and incubated for the indicated time. After 15 min, the supernatants were collected and the amounts of secreted histamine were measured by using a histamine ELISA kit according to the manufacturer’s instructions (Abnova) and by using a reader GENios Pro (Tecan). Data were expressed as nanograms per milliliter of histamine.

### MultiTox-Glo multiplex cytotoxicity assay

The relative number of live and dead cells was measured after 15 min in RBL-2H3 cells (2 × 10^5^ cells/well) stimulated with SP (10 μM) and treated with PEA (10 μM) by using MultiTox-Glo multiplex cytotoxicity kit, according to the manufacturer’s instructions (Promega Italia). Relative fluorescence units (RFU) were measured by using a GloMax Multi Detection System (Promega Italia).

### Toluidine blue staining

SP-stimulated RBL-2H3 cells [plated on poly-L-lysine (33 μg/ml) coated slides (Deckglaser, 21 × 26 mm) into 6-well culture dishes at a cell density of 9 × 10^5^ cells per well] were treated and incubated as described above for histamine release assay. After 15 min, the cells were fixed with paraformaldehyde at 4% for 20 min and incubated for 3 min with toluidine blue at 0.01% in 3% acetic acid. Subsequently, a 5 min wash in distilled water and dehydration in increasing alcohols (90%, 100%) were performed. Cells were then clarified by treatment with Xylol for 5 min and finally dried slides were mounted with the DPX histogram upright. The cells were observed using a Leica DMI6000 digital microscope, acquired using the Leica DFC 340FX digital camera connected to the microscope and analyzed using the LAS AF 2.2.0 software. Degranulated RBL-2H3 cells were counted and the percentage of degranulation (based on the number of colorable cells) was calculated.

### Measurement by LC-APCI-MS of endogenous AEA, 2-AG, and PEA levels

RBL-2H3 cells (9 × 10^5^ cells/well) were stimulated with SP (10 μM) and treated with PEA (10 μM) in the presence and absence of OMDM188 (10 μM), for 15 min at 37 °C in 5% CO_2_ atmosphere. After 15 min, cells and supernatants were collected and homogenized in a solution of CHCl_3_/CH_3_OH/Tris-HCl 50 mM pH 7.4 (2:1:1, v/v) containing 10 pmol of [^2^H]_8_-AEA, [^2^H]_5_-2-AG and [^2^H]_4_-PEA as internal standards [35]. The lipid-containing organic phase was dried down, weighed and pre-purified by open-bed chromatography on silica gel. Fractions obtained by eluting the column with a solution of CHCl_3_/CH_3_OH (90:10 by vol.) were analyzed by Liquid Chromatography-Atmospheric Pressure Chemical Ionization-Mass Spectrometry (LC-APCI-MS) using a Shimadzu (Shimadzu, Kyoto, Japan) HPLC apparatus (LC-10ADVP) coupled to a Shimadzu (LCMS-2020) quadrupole MS via a Shimadzu APCI interface. LC-APCI-MS analyses of 2-AG and PEA were carried out in the selected ion monitoring mode [19, 36], using *m/z* values of 356 and 348 (molecular ions + 1 for deuterated and undeuterated AEA), 384.35 and 379.35 (molecular ions + 1 for deuterated and undeuterated 2-AG), and 304 and 300 (molecular ions + 1 for deuterated and undeuterated PEA). AEA, 2-AG and PEA levels were calculated on the basis of their area ratio with the internal deuterated standard signal areas, and their amounts (pmol) were normalized per mg of lipid extract.

### Quantitative real-time PCR

The mRNA expression of PEA target genes (*Cnr1*, *Cnr2*, *Trpv1*, *Ppara*, and *Gpr55*), as well as PEA and 2-AG biosynthetic (*N*-acyl phosphatidylethanolamine-specific phospholipase D, *Napepld, Dagla and Daglb*) and catabolic enzyme genes (fatty acid amide hydrolase, *Faah*; *N*-acylethanolamine-hydrolyzing acid amidase, *Naaa*; and monoacylglycerol lipase, *Mgl*), was studied by comparison of transcriptional expression in unstimulated RBL-2H3 cells (plated on 100 mm diameter Petri dishes) vs*.* the expression of these targets and enzymes in RBL-2H3 cells treated with PEA (10 μM), or stimulated with SP (10 μM) in the presence and absence of PEA (10 μM), for 15 min at 37 °C in 5% CO_2_ atmosphere. Total RNA was purified, quantified and reverse transcribed as previously reported [37]. For each target, all mRNA sequences were aligned and common primers were designed (Table 1). Quantitative real-time PCR was performed by an iCycler-iQ5 in a 20 μl reaction mixture using 20 ng of cDNA. Assays were performed in quadruplicate (maximum ΔCt of replicate samples <  0.5). Optimized primers for SYBR-green analysis and optimum annealing temperatures were designed by the Allele-Id software version 7.0 (Biosoft International) and were synthesized (HPLC-purification grade) by MWG-Biotech. Relative expression calculation was corrected for PCR efficiency, normalized with respect to the reference genes β-actin and hypoxanthine phosphoribosyltransferase (HPRT) and performed by the iQ5 software. Results were expressed as fold expression compared with a reference condition (2^^−∆∆ct^ formula).

**Table 1:** List of primer sequences used in qPCR analysis

Gene	Forward sequences (5'->3')	Reverse sequences (5'->3')
*Cnr1*	CTGAGGGTTCCCTCCCGGCA	TGCTGGGACCAACGGGGAGT
*Cnr2*	GCAACTTCGTCATCTTCC	AGCACAGACATAGGTATCG
*Trpv1*	CATCTTCACTACCAGGAG	TGGATAGTTAGAACAGAGC
*Ppara*	CCTCAGGATACCACTATG	TGTTCACAGGTAAGGATT
*Gpr55*	TCTTCTGGTCAATCACTT	AATGCTCAGTAGAATGTG
*Napepld*	CGCCAAGCCATCAGTATCC	ATCCTTCTCCATTATCAGCCATC
*Dagla*	ATGATGGTGCCTGAGAGC	AGTGGGAAGGAGGGTGAG
*Daglb*	AAGGTATCCAATGTGACAGG	CAGCGATGACAATCCAACT
*Faah*	ACTGCGTGACCTCCTATC	CACAGTCAGATTCCGATGG
*Naaa*	CACTTTTGTTGGCTATGTAG	TCTCGTTCATCACCAGAA
*Mgl*	GTCCTTGCTGCCAAACTG	TCCGACTTGTTCCGAGAC
β-actin	CCAGGCATTGCTGACAGG	TGGAAGGTGGACAGTGAGG
HPRT	TTGACACTGGTAAAACAATGC	GCCTGTATCCAACACTTCG

### Competition binding assay for CB2 receptors

Membranes from Human Embryonic Kidney (HEK)-293 cells overexpressing the human recombinant CB2 receptor (*B*_max_ = 4.7 pmol/mg protein) were incubated with [^3^H]-CP-55,940 (0.084 nM/k_d_ = 0.31 nM) as the high-affinity ligand. Competition curves were performed by displacing [^3^H]-CP-55,940 with increasing concentration of PEA (0.01–10 μM), or 2-AG (0.001–100 μM) both in the absence and presence of PEA (1, 5, and 10 μM), for 90 min at 30 °C, following the procedure described by the manufacturer (Perkin Elmer, Monza, Italy), and as previously reported [38]. Non-specific binding was defined by 10 μM of WIN55,212-2 (Tocris Bioscience) as the heterologous competitor (*K*_*i*_ = 2.1 nM). Data were expressed as *K*_*i*_ (μM) and calculated by applying the Cheng-Prusoff equation to the IC_50_ values for the displacement of the bound radioligand.

### Functional activity assay at the CB2 receptors

The cAMP Hunter™ eXpress G protein-coupled receptor (GPCR) assay was performed in Chinese Hamster Ovary (CHO)-Kl cells overexpressing the human CB2 receptor. G_i_-coupled cAMP modulation was measured following the manufacturer’s protocol (DiscoverX, Fremont, CA). CHO-K1 cells overexpressing the human CB2 receptor were plated into a 96-well plate (3× 10^4^ cells/well) and incubated overnight at 37 °C in 5% CO_2_ atmosphere. The media were aspirated and replaced with 30 μl of assay buffer. Cells were incubated 30 min at 37 °C with 15 μl of 3× concentration-response solutions of 2-AG (0.01–50 μM), or PEA (10 μM), prepared in presence of cell assay buffer containing a 3× of 25 μM NKH-477 solution (a water-soluble analog of Forskolin) to stimulate adenylate cyclase and enhance basal cAMP levels. We also investigated the effect of PEA on 2-AG receptor activation by co-incubation. Therefore, cells were also incubated 30 min at 37 °C with 2-AG and PEA (10 μM) in the presence of NKH-477 to stimulate adenylate cyclase and enhance cAMP levels. Following stimulation, cell lysis, and cAMP detection were performed according to the manufacturer protocol (Promega Italia) [39]. Relative luminescence units (RLU) were measured by using a GloMax Multi Detection System (Promega Italia). Data were normalized considering the NKH-477 stimulus alone as 100% of the response. The percentage of response was calculated by using the following formula: % RESPONSE = 100% × (1-(RLU of test compound-RLU of NKH-477 positive control)/(RLU of vehicle-RLU of NKH-477 positive control).

### DAGL-α enzyme activity assay

DAGL-α enzyme activity was assessed as previously reported [40, 41] by using membrane preparations (50 μg of protein) obtained from COS-7 cells overexpressing the human recombinant DAGL-α enzyme, and 1-[^14^C]oleoyl-2-arachidonoylglycerol (1.0 mCi/mmol, 25 μM, synthesized as previously reported [40, 41], as substrate in the presence of vehicle or increasing concentrations of PEA (0.1–25 μM) in Tris-HCl 50 mM pH 7.4. After the incubation (20 min at 37 °C), lipids were extracted with two volumes of CHCl_3_/CH_3_OH (2:1, v/v). The organic extracts, lyophilized under vacuum, were used to quantify the levels of 2-AG by LC-APCI-MS (as described above), or purified by using TLC on silica on polypropylene plates eluted in CHCl_3_/CH_3_OH/NH_4_OH (85:15:0.1%, v/v) as the eluting solvent. Bands corresponding to [^14^C]-oleic acid were cut and their radioactivity was measured by using Liquid Scintillation Analyzer (TRI-carb 2100TR). Data were expressed as % of DAGL-α stimulation. To quantify the levels of 2-AG by LC-APCI-MS a non-radiolabeled 1-oleoyl-2-arachidonoylglycerol substrate was used.

### DAGL-β enzyme activity assay

DAGL-β enzyme activity was assessed by using membrane preparations (100 μg of protein) obtained from RBL-2H3 cells, and 1-[^14^C]oleoyl-2-arachidonoylglycerol (1.0 mCi/mmol, 50 μM, [40, 41]), as substrate in the presence of vehicle or increasing concentrations of PEA (1–25 μM) in Tris-HCl 50 mM pH 7.4 or in Tris-HCl 50 mM pH 7.4 and CaCl_2_ 10 mM. After the incubation (20 min at 37 °C), the protocol followed the same procedures as above reported for DAGL-α enzyme activity assay. Data were expressed as % of activity of DAGL-β.

### MGL enzyme activity assay

The 10,000×g cytosolic fractions obtained from COS-7 cells (100 μg of protein) were incubated with 2-arachidonoyl-[^3^H]-glycerol (40 Ci/mmol, St. Louis, MO, USA) diluted with non-radiolabeled 2-AG (20 μM) in the presence of vehicle or increasing concentrations of PEA (0.1-25 μM), in Tris-HCl 50 mM pH 7.4 at 37 °C for 20 min [42]. After the incubation, the amounts of [^3^H]-glycerol were measured in the aqueous phase [after extraction of the incubation mixture with 2 volumes of CHCl_3_/CH_3_OH (1:1, v/v)] by using Liquid Scintillation Analyzer (TRI-carb 2100TR).

### Statistical analysis

Each experiment was performed at least three times with triplicate groups. Data were expressed as means ± standard error of the mean (SEM). Statistical analyses were performed using GraphPad Prism software version 7.0 (GraphPad Software Inc., San Diego, CA). One-way analysis of variance (ANOVA) followed by Newman-Keuls multiple comparison test was used for analysis. *p* values < 0.05 were considered statistically significant. Figures were generated in GraphPad Prism software version 7.0.

## Results

### PEA reduces β-hexosaminidase and histamine release from SP-stimulated RBL-2H3 cells

**Fig. 1:**
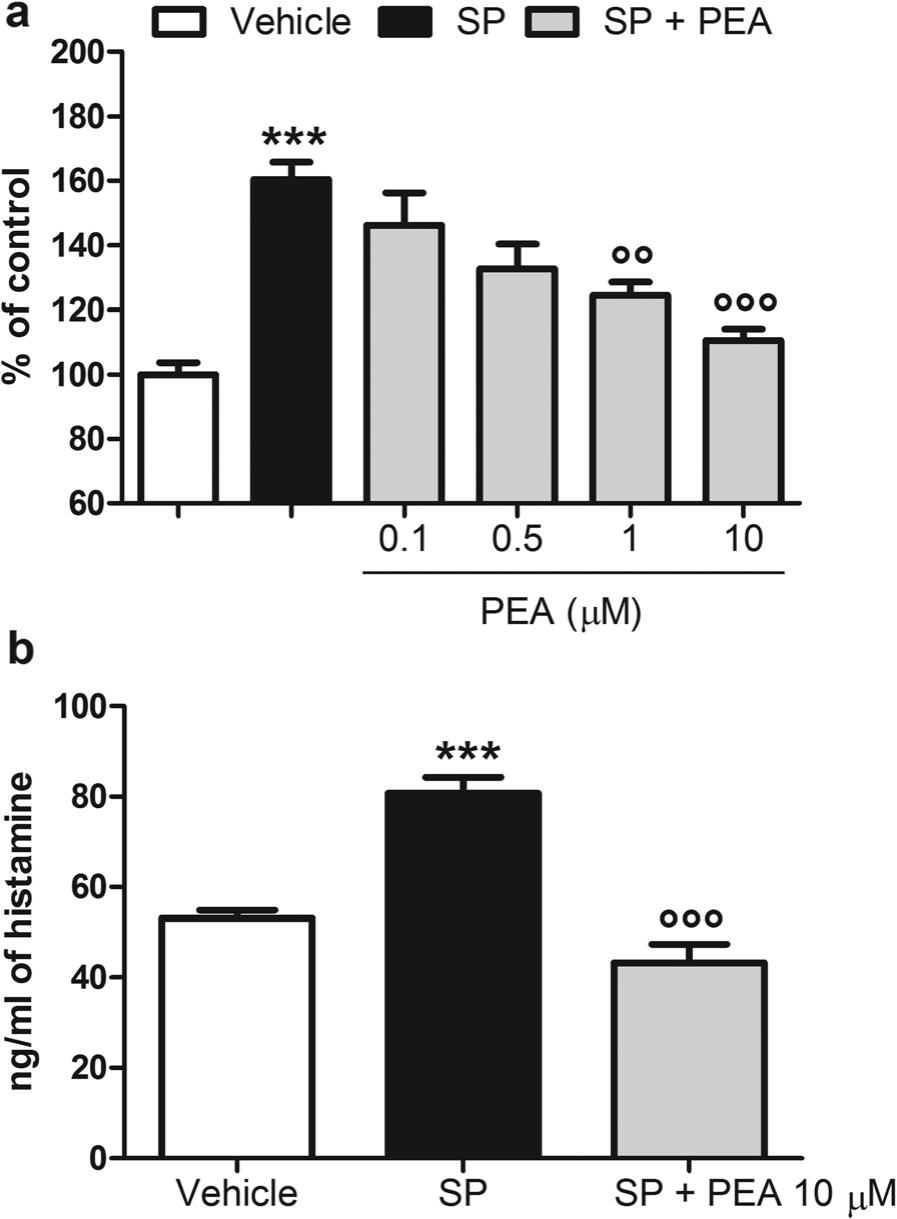
PEA reduces β-hexosaminidase and histamine release from SP-stimulated RBL-2H3 cells. **a** β-hexosaminidase release was measured after stimulation of RBL-2H3 cells with SP (10 μM) in the presence or absence of PEA (0.1, 0.5, 1, and 10 μM) for 15 min at 37 °C in a 5% CO_2_ atmosphere. Absorbance was measured at 405 nm. Each bar shows the mean ± SEM. ***p < 0.001 compared with Vehicle. °°*p* < 0.01 and °°°*p* < 0.001 compared with SP. **b** Histamine release by ELISA was performed after stimulation of RBL-2H3 cells with SP (10 μM) in the presence or absence of PEA (10 μM), for the indicated time. Absorbance was measured at 450 nm. Each bar shows the mean ± SEM. ***p < 0.001 compared with vehicle. °°°*p* < 0.001 compared with SP

RBL-2H3 cells stimulated with SP (10 μM for 15 min) and treated with the vehicle of PEA significantly released β-hexosaminidase and histamine, as compared to vehicle-stimulated RBL-2H3 cells (Fig. 1a, b). PEA (0.1, 0.5, 1, and 10 μM), in a concentration-dependent manner, strongly reduced the release of β-hexosaminidase from SP-stimulated RBL-2H3 cells, as compared to SP-stimulated RBL-2H3 cells treated with the vehicle of PEA (Fig. 1a). The maximum effect was observed at the highest concentration tested of PEA (10 μM) (Fig. 1a), which also inhibited the release of histamine from SP-stimulated RBL-2H3 cells, as compared to SP-stimulated RBL-2H3 cells treated with the vehicle of PEA (Fig. 1b). No effect on β-hexosaminidase and histamine release was observed if RBL-2H3 cells were treated with PEA alone (10 μM), i.e., in the absence of SP, as compared to vehicle-treated RBL-2H3 cells (data not shown).

### PEA does not affect the viability and cytotoxicity of both unstimulated and SP-stimulated RBL-2H3 cells

**Fig. 2:**
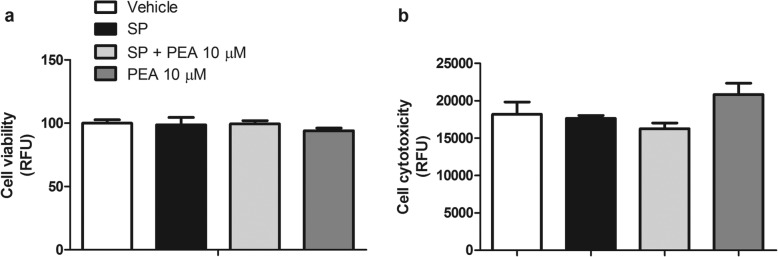
Effect of PEA on cell viability and cytotoxicity of both unstimulated and SP-stimulated RBL-2H3 cells. **a**, **b** Cell viability and cytotoxicity were assessed, by means of a MultiTox-Glo assay after that RBL-2H3 cells were treated with PEA (10 μM) or stimulated with SP (10 μM) in the presence or absence of PEA (10 μM) for 15 min at 37 °C in a 5% CO_2_ atmosphere. RFU was measured at 495 nm and 505 nm (**a**). RFU was measured at 500 nm and 550 nm (**b**). Each bar shows the mean ± SEM

No effect on viability and cytotoxicity was observed after stimulation of RBL-2H3 cells with SP (10 μM for 15 min) and the vehicle of PEA, as compared to vehicle-stimulated RBL-2H3 cells (Fig. 2a, b). Likewise, PEA (10 μM) did not alter the viability and cytotoxicity of SP-stimulated RBL-2H3 cells, as compared to vehicle-stimulated RBL-2H3 cells (Fig. 2a, b). No effect on viability and cytotoxicity was also observed when RBL-2H3 cells were treated with PEA alone (10 μM), i.e., in the absence of SP, as compared to vehicle-treated RBL-2H3 cells (Fig. 2a, b).

### A CB2 receptor antagonist blocks the effect of PEA on histamine release from SP-stimulated RBL-2H3 cells

**Fig. 3:**
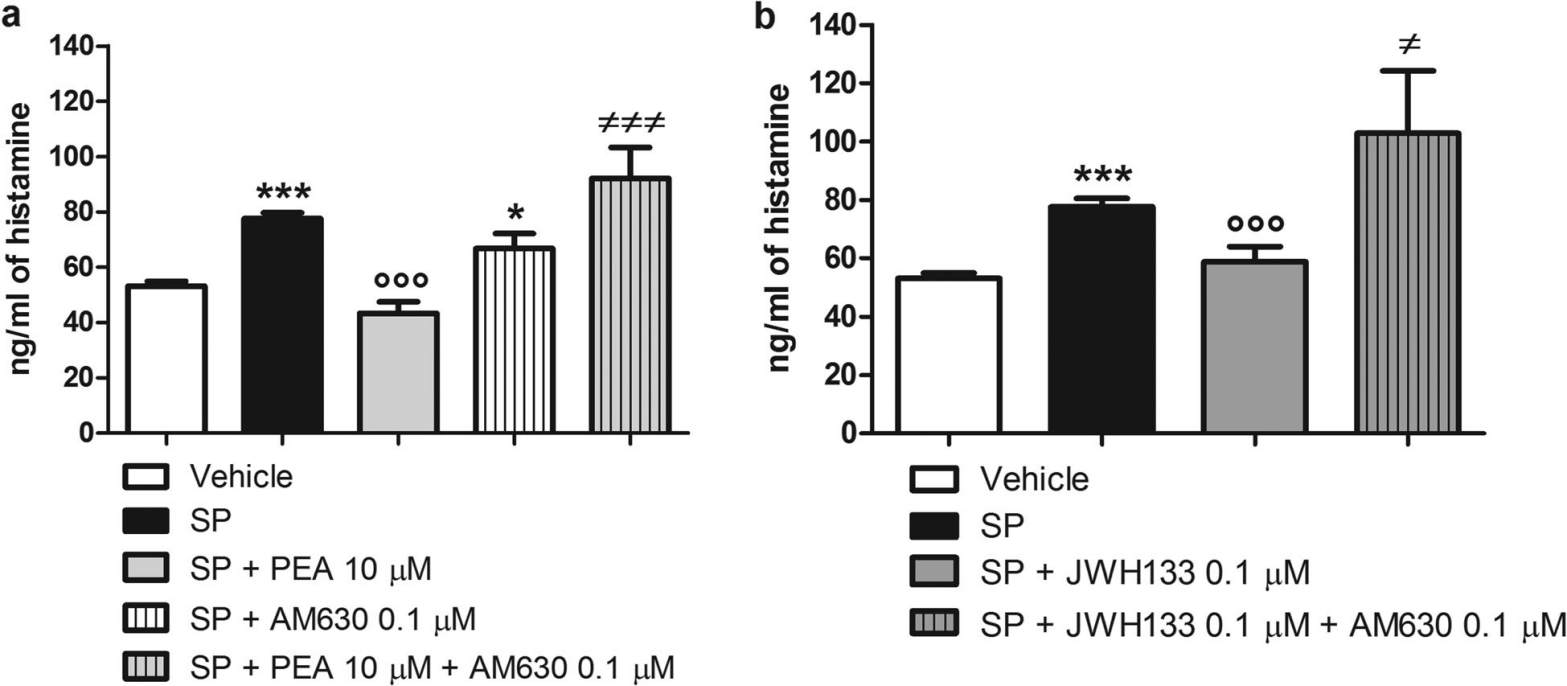
PEA and JWH133 control SP-induced histamine release in RBL-2H3 cells via a CB2-mediated mechanism. Histamine release by ELISA was performed after that RBL-2H3 cells were stimulated with SP (10 μM) and treated with AM630 (0.1 μM) in the presence or absence of **a** PEA (10 μM) or **b** JWH133 (0.1 μM), for 15 min at 37 °C in a 5% CO_2_ atmosphere. Absorbance was measured at 450 nm. Each bar shows the mean ± SEM. *p < 0.05 and ***p < 0.001 compared with Vehicle. °°°*p* < 0.001 compared with SP. ^≠≠≠^*p* < 0.001 compared with SP + PEA 10 μM. ^≠^*p* < 0.01 compared with SP +JWH133 0.1 μM

When RBL-2H3 cells were stimulated with SP (10 μM for 15 min) and treated with a selective (AM630) CB2 receptor antagonist (at the concentration of 0.1 μM), histamine release was comparable to that observed in SP-stimulated RBL-2H3 cells treated with the vehicle (Fig. 3a). Interestingly, when SP-stimulated RBL-2H3 cells were co-treated with PEA (10 μM) and AM630 (0.1 μM), histamine release was comparable to that observed in SP-stimulated RBL-2H3 cells treated with the vehicle, or with AM630 (0.1 μM) (Fig. 3a). No effect was observed on histamine release when RBL-2H3 cells were treated with the antagonist alone (data not shown).

### A synthetic CB2 agonist inhibits histamine release from SP-stimulated RBL-2H3 cells

JWH133 (0.1 μM), a synthetic CB2 receptor agonist, inhibited the release of histamine from SP-stimulated RBL-2H3 cells, as compared to SP-stimulated RBL-2H3 cells treated with the vehicle (Fig. 3b). When SP-stimulated RBL-2H3 cells were co-treated with JWH133 (0.1 μM) and AM630 (0.1 μM), histamine release was comparable to that observed in SP-stimulated RBL-2H3 cells treated with the vehicle (Fig. 3b). No effect was observed on histamine release when RBL-2H3 cells were treated with JWH133 alone (0.1 μM), i.e., in the absence of SP (data not shown).

### PEA and JWH133 downmodulate SP-induced degranulation of RBL-2H3 cells via a CB2-mediated mechanism

**Fig. 4:**
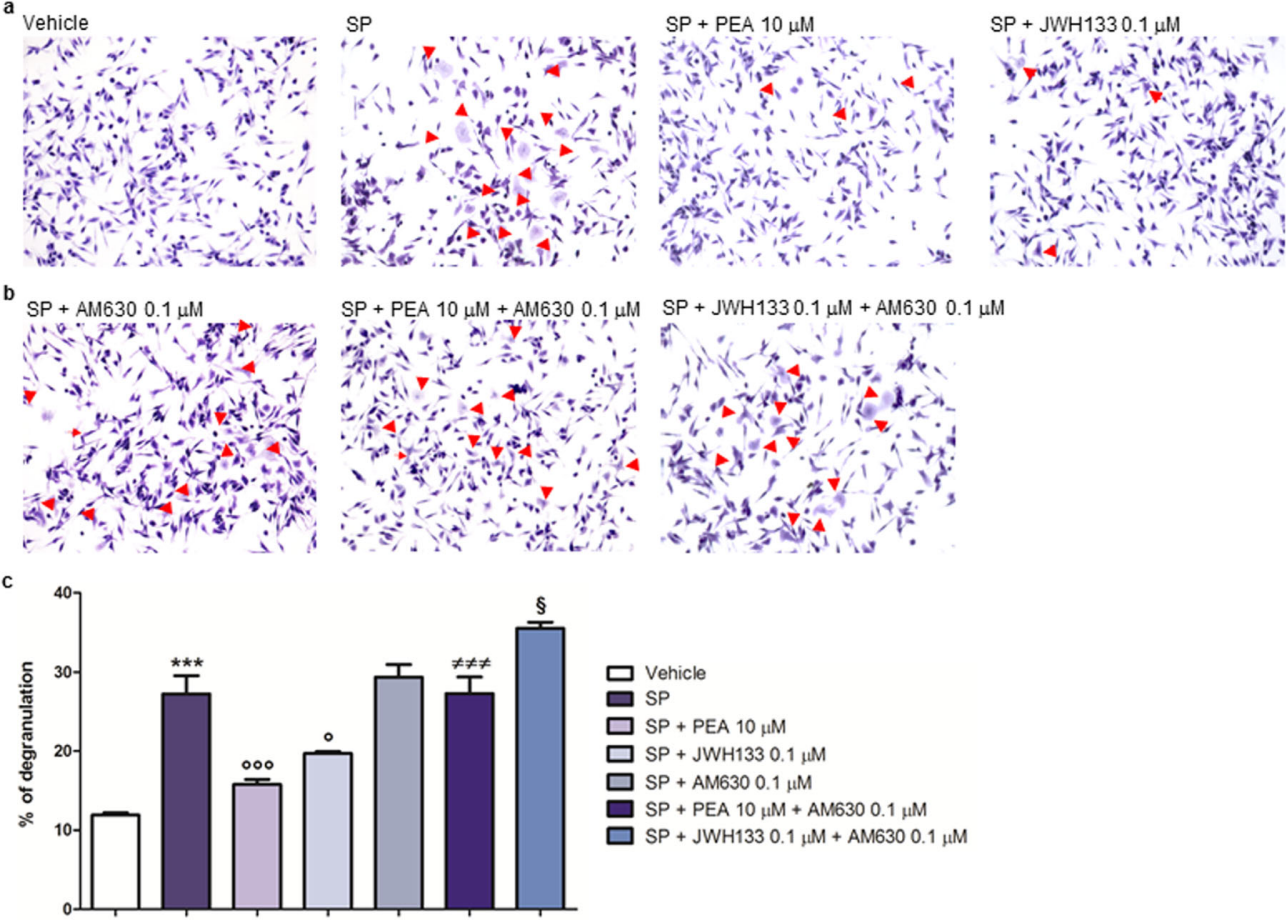
PEA and JWH133 down-modulate SP-induced degranulation of RBL-2H3 cells via a CB2-mediated mechanism. Toluidine blue staining was performed to measure the number of degranulated RBL-2H3 cells after that **a** RBL-2H3 cells were stimulated with SP (10 μM) in the presence and absence of PEA (10 μM), or JWH133 (0.1 μM), for 15 min at 37 °C in a 5% CO_2_ atmosphere; **b** SP-stimulated RBL-2H3 cells were treated with AM630 (0.1 μM), in the presence and absence of PEA (10 μM), or JWH133 (0.1 μM), for the indicated time. Red arrows show degranulated RBL-2H3 cells. **c** Percentage of degranulation. Each bar shows the mean ± SEM. ***p < 0.001 compared with vehicle. °*p* < 0.05 and °°°*p* < 0.001 compared with SP. ^≠≠≠^*p* < 0.001 compared with SP + PEA 10 μM. ^§^*p* < 0.05 compared with SP + JWH133 0.1 μM

SP (10 μM for 15 min) increased the number of degranulated RBL-2H3 cells, as compared to vehicle-stimulated cells (Fig. 4a, c). PEA (10 μM) reduced the number of SP-degranulated RBL-2H3 cells, as compared to SP-stimulated RBL-2H3 cells treated with the vehicle (Fig. 4a, c). When SP-stimulated RBL-2H3 cells were treated with AM630 (0.1 μM), the number of degranulated RBL-2H3 cells was comparable to that measured in SP-stimulated RBL-2H3 cells treated with the vehicle, i.e., in the absence of the antagonist (Fig. 4a–c). More importantly, when SP-stimulated RBL-2H3 cells were co-treated with PEA (10 μM) and AM630 (0.1 μM), the number of degranulated RBL-2H3 cells was again comparable to that measured in SP-stimulated RBL-2H3 cells treated with the vehicle, i.e., in the absence of both PEA and the antagonist (Fig. 4a–c), or with the antagonist, i.e., in the absence of PEA (Fig. 4b, c). In addition, we observed that JWH133 (0.1 μM), similar to PEA (10 μM), also reduced the number of SP-degranulated RBL-2H3 cells, as compared to SP-stimulated RBL-2H3 cells treated with the vehicle (Fig. 4a, c), and its effect was reversed by AM630 (0.1 μM) (Fig. 4a–c). In fact, the number of SP-degranulated RBL-2H3 cells following co-treatment with JWH133 (0.1 μM) and AM630 (0.1 μM) was comparable to that measured in SP-stimulated RBL-2H3 cells treated only with the vehicle (Fig. 4a–c), or only with the antagonist (Fig. 4b, c). Finally, no effect was observed on degranulation when RBL-2H3 cells were treated with PEA (10 μM) or JWH133 (0.1 μM) alone, i.e., in the absence of SP (data not shown).

### PEA increases the levels of 2-AG in both unstimulated and SP-stimulated RBL-2H3 cells

**Fig. 5:**
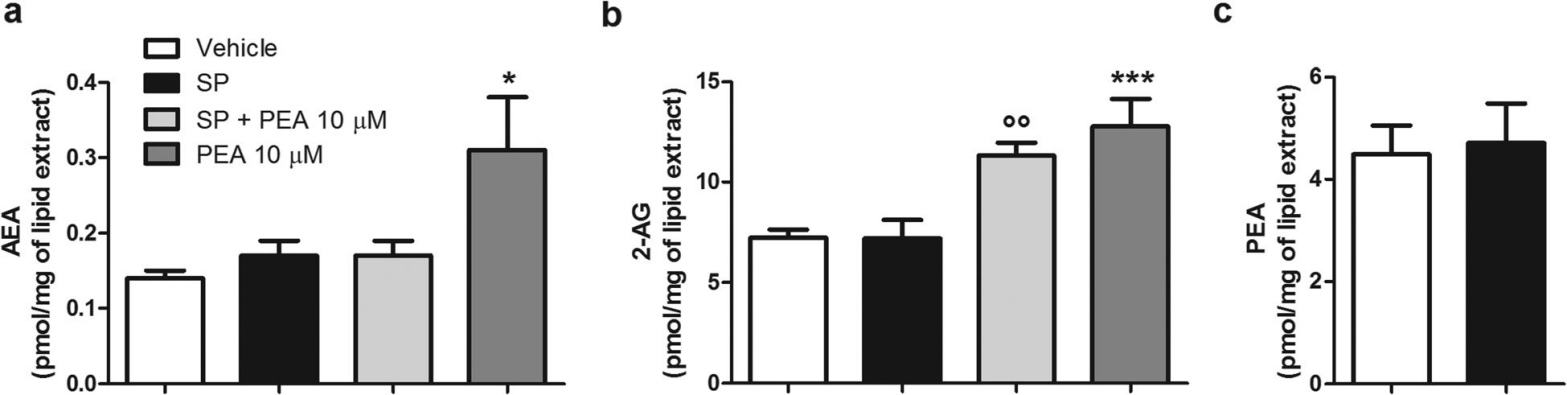
PEA increases 2-AG levels in either unstimulated or SP-stimulated RBL-2H3 cells. **a–c** AEA, 2-AG, and PEA levels were quantified, by LC-MS, after that RBL-2H3 cells were treated with PEA (10 μM) or stimulated with SP (10 μM) in the presence or absence of PEA (10 μM) for 15 min at 37 °C in a 5% CO_2_ atmosphere. Each bar shows the mean ± SEM. *p < 0.05 and ***p < 0.001 compared with vehicle. °°*p* < 0.01 compared with SP

When RBL-2H3 cells were stimulated with SP under the same conditions shown above to induce preformed mediator release and degranulation (10 μM for 15 min), the endogenous levels of AEA, 2-AG, and PEA did not change, as compared to RBL-2H3 cells stimulated with vehicle (Fig. 5a–c). By contrast, when SP-stimulated RBL-2H3 cells were treated with PEA (10 μM), the endogenous levels of 2-AG were significantly increased by 1.4-fold compared to RBL-2H3 cells only treated with vehicle (Fig. 5b), and by 1.6-fold compared to SP-stimulated RBL-2H3 cells treated with the PEA vehicle (Fig. 5b). In addition, the endogenous levels of 2-AG were also significantly increased by 1.8-fold when RBL-2H3 cells were treated with PEA (10 μM) alone, i.e., in the absence of SP, as compared to vehicle-treated RBL-2H3 cells (Fig. 5b). It is noteworthy that, considering that 1 mg of lipids are usually extracted from 10 mg of cell pellet (*personal communication by Petrosino S and Di Marzo V*), i.e., a volume of 10 μl, the concentration of 2-AG in SP-stimulated RBL-2H3 cells treated with PEA (10 μM) can be estimated to be about 1.2 μM *vs.* 0.7 μM in unstimulated cells, pointing to an increase of 0.5 μM, which is sufficient to fully activate CB2. Finally, no statistically significant increase of the endogenous levels of AEA was observed when SP-stimulated RBL-2H3 cells were treated with PEA (10 μM), as compared to SP-stimulated RBL-2H3 cells treated with PEA vehicle (Fig. 5a). In contrast, a statistically significant increase of the endogenous levels of AEA was observed when unstimulated RBL-2H3 cells were treated with PEA alone (10 μM), as compared to vehicle-treated RBL-2H3 cells (Fig. 5a).

### PEA does not modulate the mRNA expression of its targets, nor that of its or 2-AG biosynthetic and catabolic enzymes

**Fig. 6:**
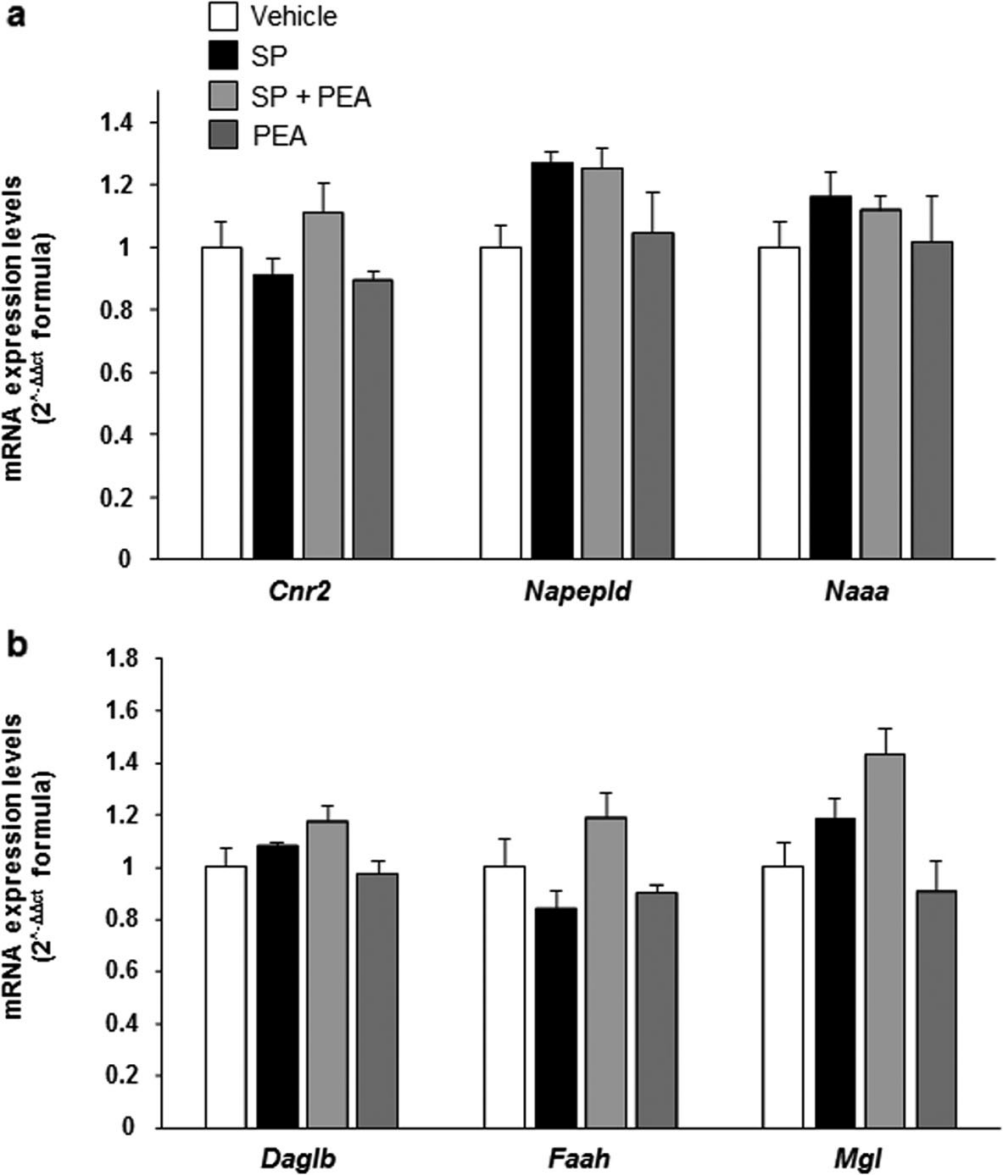
Effect of PEA on mRNA expression levels of PEA and 2-AG receptors and metabolic enzymes. Real-time qPCR analysis showing the transcript levels of **a** *Cnr2*, *Napepld*, and *Naaa*; and **b** *Daglb*, *Faah*, and *Mgl*, in RBL-2H3 cells treated with PEA (10 μM) or stimulated with SP (10 μM) in the presence or absence of PEA (10 μM), for 15 min at 37 °C in a 5% CO_2_ atmosphere. Each bar shows the mean ± SEM

In unstimulated RBL-2H3 cells, we found a robust mRNA expression of *Napepld* and *Naaa* (Fig. 6a, b), whereas less robust mRNA expression of *Cnr2*, *Daglb*, *Faah*, and *Mgl* (Fig. 6a, b) was found (Table 2). RBL-2H3 cells stimulated (for 15 min) with either SP (10 μM) or PEA (10 μM) or both showed no statistically significant change in the expression of the mRNAs encoding for these receptors and enzymes (Fig. 6a, b). A very low expression of *Cnr1* and no expression of *Trpv1*, *Ppara*, *Gpr55*, and *Dagla* was found in either unstimulated or SP-stimulated RBL-2H3 cells, treated or untreated with PEA (data not shown).

**Table 2:** mRNA expression levels of PEA and 2-AG receptors and metabolic enzymes

Gene	CT values ± SD
*Cnr1*	30.95 ± 0.39
*Cnr2*	26.45 ± 0.22
*Trpv1*	N.D.
*Ppara*	N.D.
*Gpr55*	N.D.
*Napepld*	23.76 ± 0.09
*Dagla*	N.D.
*Daglb*	26.98 ± 0.10
*Faah*	26.91 ± 0.21
*Naaa*	24.30 ± 0.12
*Mgl*	26.77 ± 0.09
β-actin	14.98 ± 0.15
HPRT	21.91 ± 0.18

### Lack of significant effects of PEA on the binding and functional activity of 2-AG at the human recombinant CB2 receptor

**Fig. 7:**
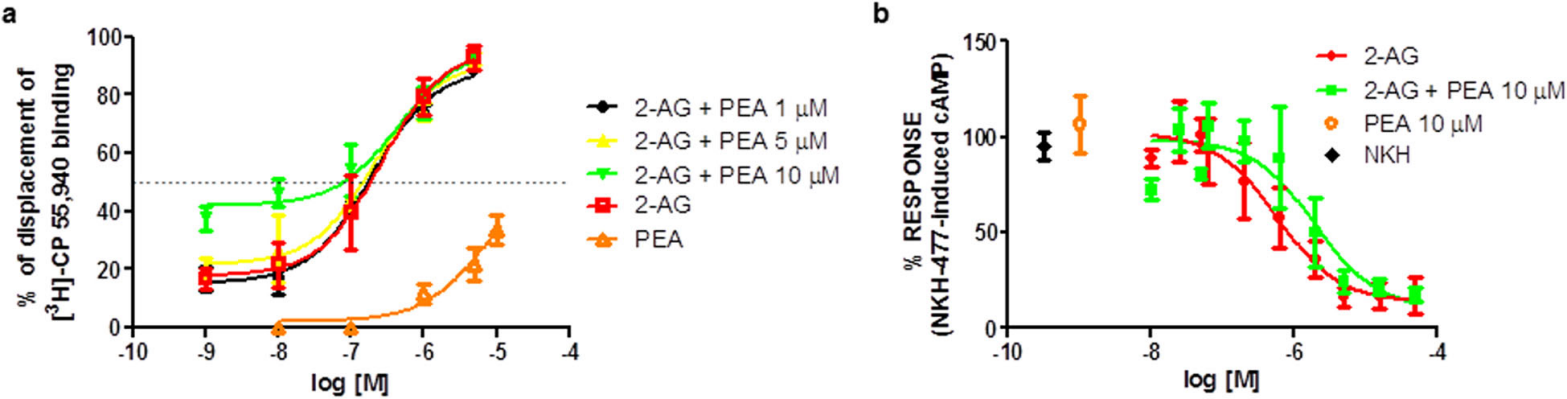
Effects of PEA on 2-AG affinity and efficacy at the human CB2 receptor. **a** Displacement curves of 2-AG and PEA, alone and in combination, in a competition binding assay. The curves show the effect of increasing concentrations of 2-AG, PEA, or 2-AG plus PEA at displacing [^3^H]-CP-55,940 from the human recombinant CB2. All experiments were performed in membranes from HEK-293 cells overexpressing the human recombinant CB2 receptors. Data are the mean ± SEM. The effect of WIN55, 212-2 (10 μM) was considered as 100% displacement. **b** Concentration-response curves of 2-AG and PEA, alone and in combination, in a cAMP-based functional assay. The curves show the % of the response relative to the maximum effect observed on NKH-477-induced cAMP levels in CHO-Kl cells stably overexpressing the human recombinant CB2 receptor with increasing concentrations of 2-AG, PEA, or 2-AG following incubation with PEA.

Binding data indicated that 2-AG alone showed high-binding affinity for CB2 (*K*_*i*_ = 0.07 ± 0.01 μM) (Fig. 7a), whereas PEA alone did not show a measurable affinity for this receptor (*K*_*i*_ > 10 μM) (Fig. 7a). When 2-AG was co-incubated with the two lowest concentrations tested of PEA (1 and 5 μM), its binding affinity did not statistically change (*K*_*i*_ = 0.06 ± 0.01 and 0.07 ± 0.01 μM, respectively) (Fig. 7a). However, when 2-AG was incubated with the highest concentration tested of PEA (10 μM), we found a significant improvement of its binding affinity (*K*_*i*_ = 0.02 ± 0.005 μM) (Fig. 7a), which however appeared to be due to the little effect on [^3^H]-CP55,940 displacement exerted per se by PEA (10 μM) (33.51 ± 5.28%) (Fig. 7a).

PEA did not activate CB2 since, at the highest concentration tested (10 μM), it failed at decreasing cAMP levels under the stimulus of NKH-477 (Fig. 7b). On the contrary, 2-AG in a concentration-dependent manner reduced NKH-477-induced cAMP levels (IC_50_ = 590 ± 160 nM). The presence of PEA (10 μM) slightly decreased 2-AG efficacy (IC_50_ = 1988 ± 220 nM), although in a non-statistically significant manner, and enhanced the effect only of the lowest concentration of 2-AG tested (10 nM) (Fig. 7b).

### PEA stimulates the activity of DAGL-α and -β and the biosynthesis of 2-AG in COS-7 cells over-expressing DAGL-α

**Fig. 8:**
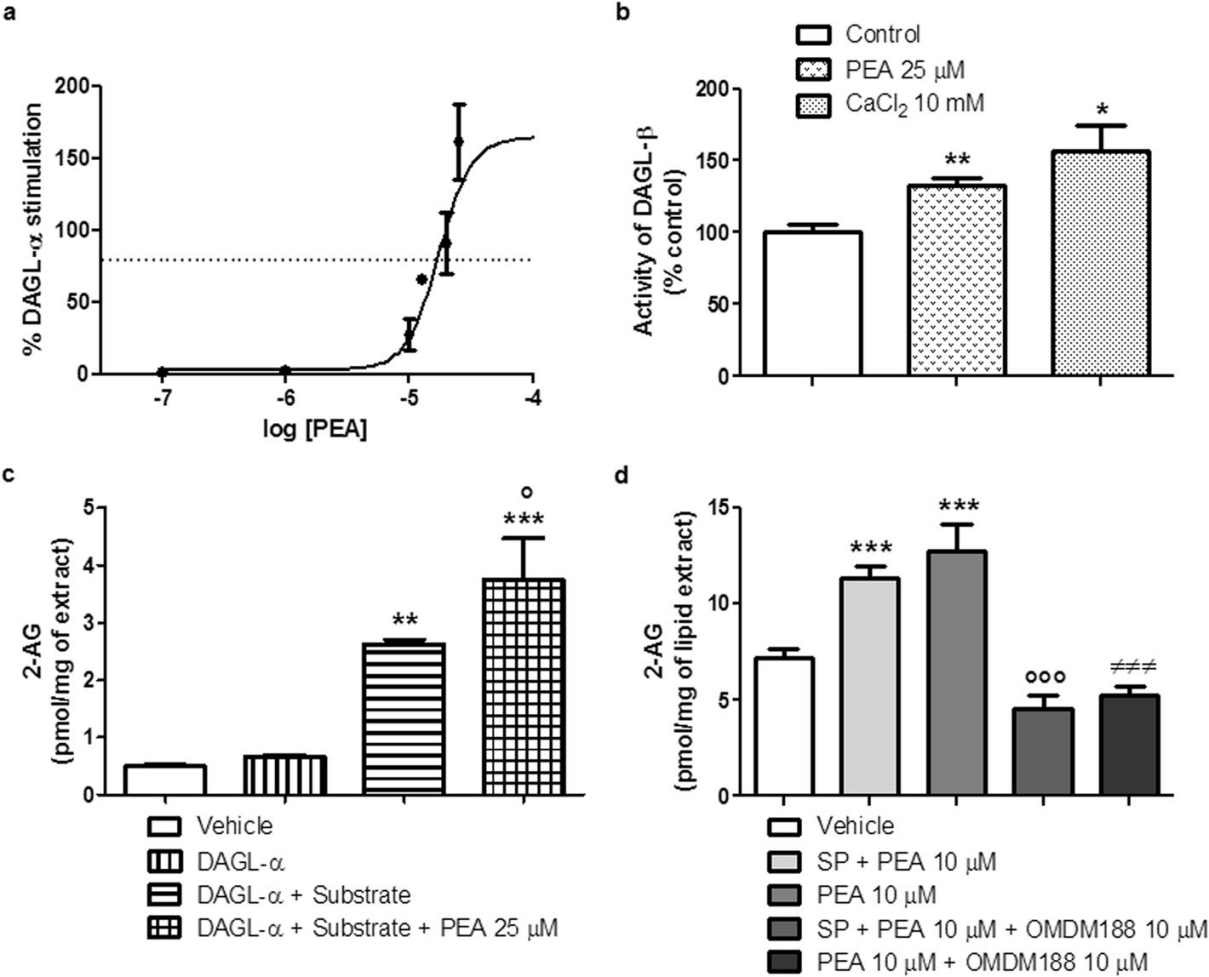
PEA stimulates DAGL-α and -β. **a** Concentration-response curve for the stimulation of DAGL-α activity by PEA. The curve shows the % of stimulation compared to the activity of the enzyme with no PEA, observed with increasing concentrations of PEA in membranes obtained from COS-7 cells over-expressing human recombinant DAGL-α. Data are the means ± SEM. **b** Effect of PEA (25 μM) and CaCl_2_ (10 mM) on DAGL-β activity in RBL-2H3 cell membranes. Data are the means ± SEM. *p < 0.05 and **p < 0.01 compared with Control. **c** The levels of 2-AG by LC-MS were measured after that membrane preparations (70 μg of protein) from COS-7 cells over-expressing DAGL-α were incubated with 1-oleoyl-2-arachidonoylglycerol (25 μM) in the presence or absence of PEA (25 μM) for 20 min at 37 °C, i.e., using the same conditions for enzyme activity assay as in **a**. Each bar shows the mean ± SEM. **p < 0.01 and ***p < 0.001 compared with DAGL-α. °*p* < 0.05 compared with DAGL-α + substrate. **d** The endogenous levels of 2-AG were measured after that RBL-2H3 cells were treated with PEA (10 μM) in the presence or absence of the DAGL inhibitor, OMDM188 (10 μM), or stimulated with SP (10 μM) and treated with PEA (10 μM) in the presence or absence of OMDM188 (10 μM), for 15 min at 37 °C in a 5% CO_2_ atmosphere. Each bar shows the mean ± SEM. ***p < 0.001 compared with vehicle. °°°*p* < 0.001 compared with SP + PEA 10 μM. ^≠≠≠^*p* < 0.001 compared with PEA 10 μM. Vehicle, SP + PEA 10 μM and PEA 10 μM data are the same as in Fig. 5b

PEA stimulated DAGL-α activity with an EC_50_ value of 17.3 ± 2.35 μM (Fig. 8a), in COS-7 cells over-expressing DAGL-α. PEA also stimulated the activity of DAGL-β by 33 ± 5.43% at the concentration of 25 μM (Fig. 8b), in RBL-2H3 cells. Importantly, the stimulatory effect of PEA (25 μM) on RBL-2H3 cell DAGL-β activity was comparable to that observed with Ca^2+^ (10 mM) (Fig. 8b). Instead, PEA exhibited no inhibitory effect on MGL activity up to 25 μM (the maximal % inhibition was calculated to be < 5%).

We also measured by LC-MS the levels of 2-AG produced after enzymatic hydrolysis of the substrate 1-oleoyl-2-arachidonoylglycerol by DAGL-α, in the presence or absence of PEA (25 μM). The analysis revealed that when membrane preparations obtained from COS-7 cells over-expressing DAGL-α were incubated with the substrate 1-oleoyl-2-arachidonoylglycerol, the levels of 2-AG were significantly increased by 3.9-fold compared to membrane preparations incubated in the absence of the substrate (Fig. 8c). PEA (25 μM) was able to significantly further elevate the levels of 2-AG: i) by 1.4-fold compared to membrane preparations incubated with the substrate and without PEA; and ii) by 5.6-fold compared to membrane preparations incubated alone, i.e., in the absence of both the substrate and PEA (Fig. 8c).

### OMDM188 blocks the stimulatory effect of PEA on 2-AG levels in both untreated and SP-treated RBL-2H3 cells

The analysis by LC-MS revealed that when SP-stimulated RBL-2H3 cells (10 μM for 15 min) were treated with OMDM188 (10 μM), a DAGL inhibitor [43], in the presence of PEA (10 μM), the endogenous levels of 2-AG were decreased by 2.5-fold compared to SP-stimulated RBL-2H3 cells treated only with PEA (Fig. 8d). Likewise, when unstimulated RBL-2H3 cells were treated with OMDM188 (10 μM) in the presence of PEA (10 μM), the endogenous levels of 2-AG were decreased by 2.4-fold compared to unstimulated RBL-2H3 cells treated with PEA (10 μM) alone (Fig. 8d).

### OMDM188 blocks the effect of PEA on SP-induced histamine release and degranulation in RBL-2H3 cells

**Fig. 9:**
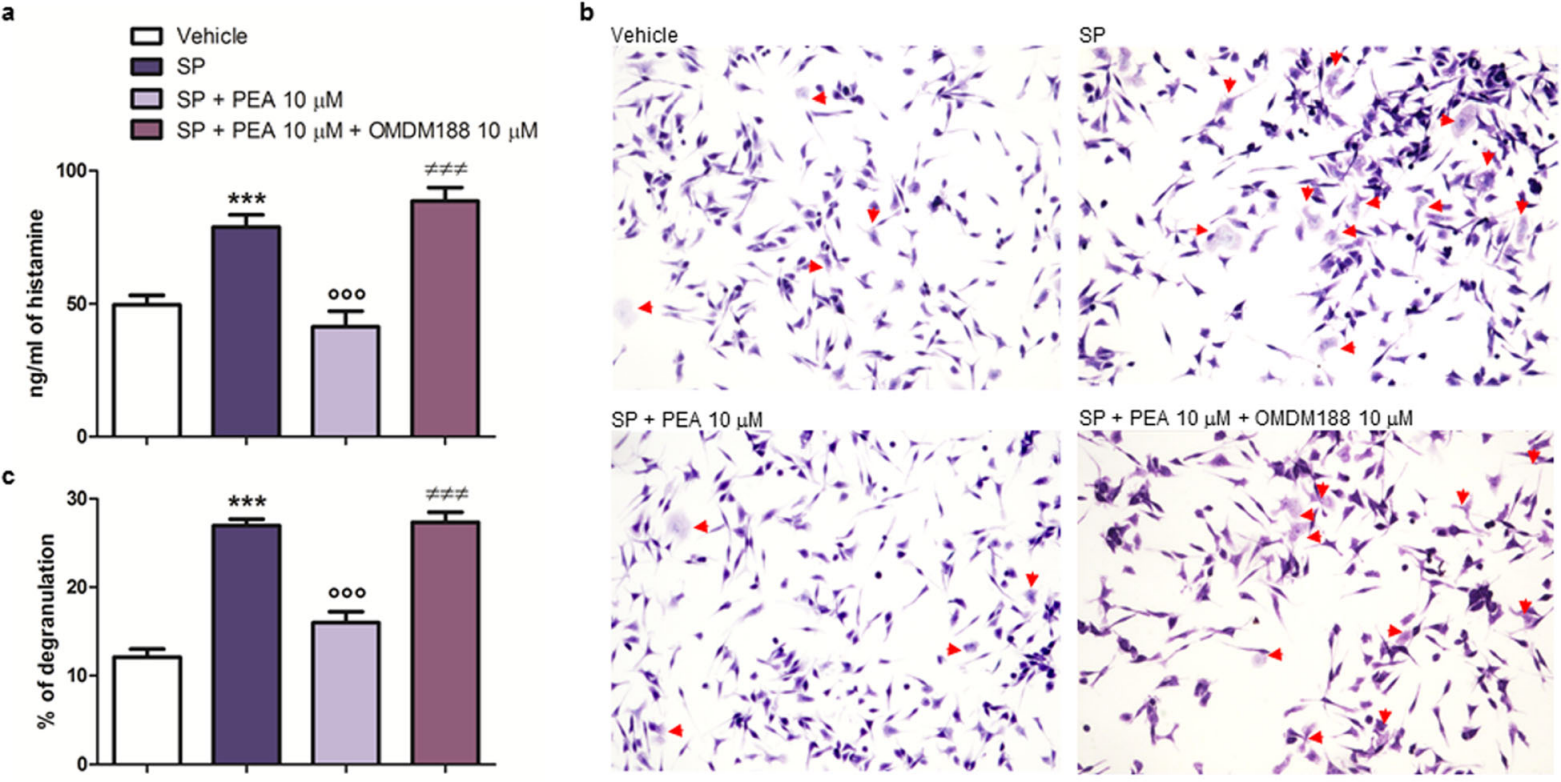
OMDM188 blocks PEA down-modulation of SP-induced histamine release and degranulation in RBL-2H3 cells. **a** Histamine release by ELISA, **b** Toluidine blue staining, and **c** the percent of degranulation were measured after that RBL-2H3 cells were stimulated with SP (10 μM) and treated with PEA (10 μM) in the presence or absence of OMDM188 (10 μM) for 15 min at 37 °C in a 5% CO_2_ atmosphere. Absorbance was measured at 450 nm (**a**). Red arrows show degranulated RBL-2H3 cells (**b**). Each bar (**a**, **c**) shows the mean ± SEM. ***p < 0.001 compared with vehicle. °°°*p* < 0.001 compared with SP. ^≠≠≠^*p* < 0.001 compared with SP + PEA 10 μM

When SP-stimulated RBL-2H3 cells (10 μM for 15 min) were treated with OMDM188 (10 μM) in the presence of PEA (10 μM), histamine release (Fig. 9a) and the number of degranulated RBL-2H3 cells (Fig. 9b, c) were comparable to that observed in SP-stimulated RBL-2H3 cells treated with the vehicle, i.e., in the absence of both OMDM188 and PEA (Fig. 9).

### PEA and 2-AG synergize at down-modulating SP-induced histamine release and degranulation in RBL-2H3 cells

**Fig. 10:**
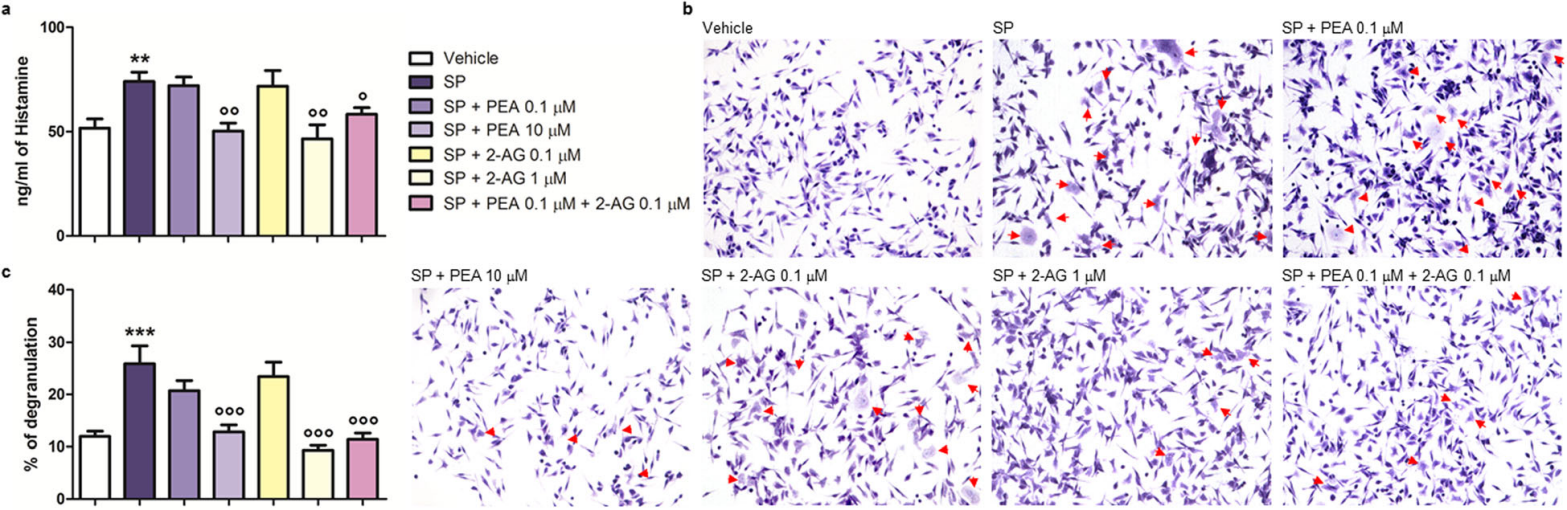
Co-treatment with subeffective concentrations of PEA and 2-AG down-modulates SP-induced histamine release and degranulation in RBL-2H3 cells. **a** Histamine release by ELISA, **b** Toluidine blue staining, and **c** the percent of degranulation were measured after that RBL-2H3 cells were stimulated with SP (10 μM) and treated with PEA (0.1 and 10 μM), 2-AG (0.1 and 1 μM), or PEA (0.1 μM) plus 2-AG (0.1 μM), for 15 min at 37 °C in a 5% CO_2_ atmosphere. Absorbance was measured at 450 nm (**a**). Red arrows show degranulated RBL-2H3 cells (**b**). Each bar (**a**, **c**) shows the mean ± SEM. **p < 0.01 and ***p < 0.001 compared with vehicle. °*p* < 0.05, °°*p* < 0.01, and °°°*p* < 0.001 compared with SP

When SP-stimulated RBL-2H3 cells (10 μM for 15 min) were treated with either PEA or 2-AG, both at the lowest concentration tested (0.1 μM), histamine release (Fig. 10a) and degranulation (Fig. 10b, c) were comparable to that observed in SP-stimulated RBL-2H3 cells treated only with the vehicle (Fig. 10). By contrast, 2-AG at the highest concentration tested (1 μM), like PEA (10 μM), was able to reduce SP-induced histamine release (Fig. 10a) and degranulation (Fig. 10b, c) in RBL-2H3 cells, as compared to SP-stimulated RBL-2H3 cells treated only with vehicle (Fig. 10). Co-treatment with PEA and 2-AG, both at the per se ineffective concentration of 0.1 μM, was able to reduce the release of histamine (Fig. 10a) and the number of degranulated cells (Fig. 10b, c) from SP-stimulated RBL-2H3 cells, as compared to SP-stimulated RBL-2H3 cells treated with the highest concentration tested of PEA (10 μM) or 2-AG (1 μM) (Fig. 10).

## Discussion

NI is a well-known process participating in the pathogenesis of several diseases of the nervous and respiratory systems, gastrointestinal and urogenital tracts, and skin [44]. It is elicited by the release of potent pro-algesic and inflammatory mediators, among which the neuropeptides SP and calcitonin gene-related peptide, from sensory nerve fibers (particularly C-fibers) afferent to the skin, and respiratory, intestinal and urinary tissues [44]. Once released, the neuropeptides trigger a cascade of inflammatory responses including the degranulation of adjacent MC, and hence the release of pre-formed mediators, among which histamine, from MC granules [44]. MC are a key player in the immune system exerting both a regulatory role, in as much as they are capable of suppressing the inflammatory processes [45], and an effector role when deregulated, such as, for example, during NI, when they exacerbate the progression of the inflammatory disease [45]. NI is currently viewed as a common substrate for different diseases [46–49].

PEA, a lipid produced *on demand* in many animal cells and tissues, acts as a balancer in those disorders associated with neuroinflammation by suppressing the pathological consequences triggered by over-stimulated MC [2, 4, 9, 10]. In fact, PEA is able to downmodulate MC activation and degranulation by reducing the release of β-hexosaminidase and serotonin induced by IgE receptor crosslinking in RBL-2H3 cells [27, 50], as well as the amount of degranulation and subsequent plasma extravasation induced by SP injection in the mouse ear pinna [29]. CB2 receptors were initially suggested to be involved in most of these effects of PEA, which, accordingly, were attenuated by the CB2 antagonist SR144528 [27, 51], as were other reported anti-inflammatory and analgesic actions of this lipid [52, 53]. Later, it was clearly demonstrated that PEA exhibits only very weak activity at CB2 receptors [12], and as a result several hypotheses on its mechanism of action were developed [2, 4]. One of these is known as *entourage effect* and had been earlier on proposed to underlie also the cannabimimetic effect of non-cannabinoid receptor active monoacylglycerol homologs of 2-AG [17]. It consists in the capability of PEA to potentiate the signaling of endocannabinoids and endovanilloids at CB1 and CB2 receptors or TRPV1 channels, through several receptor- (PPARα, GPR55) and non-receptor-mediated mechanisms, and has gained increasing evidence over the last 20 years [13, 18–20, 22, 23, 25, 26]. Nevertheless, before the present study, the entourage effect of PEA had never been extended to the very first reported example of PEA anti-inflammatory effects, i.e., its capability of downregulating MC hyperactivity [27]. Here we demonstrate for the first time that this very important protective action of PEA, described here to occur also in what could be considered a simplified in vitro model of NI, is due to a direct stimulatory effect of the lipid on 2-AG biosynthesizing enzymes, the DAGLs α and β, and the subsequent elevation of cellular 2-AG concentrations.

We used the widely employed RBL-2H3 cell line as a MC model. Indeed, following incubation with SP, these cells undergo degranulation and released β-hexosaminidase and histamine into the extracellular medium. In agreement with its previously described MC stabilizing effect [27], we first found that PEA dose-dependently down-modulates SP-induced degranulation of RBL-2H3 cells and the release therefrom of β-hexosaminidase and histamine. We then assigned these effects of PEA exclusively to its ability to reduce the response to SP stimulation, inasmuch as we showed that neither SP stimulation nor PEA treatment affected the viability and cytotoxicity of RBL-2H3 cells. More importantly, we confirmed that these effects were due to activation of CB2, as shown not only by the fact that they were blocked by a selective CB2 receptor antagonist, used at a concentration selective vs. CB1 receptors, but also by the finding that the synthetic CB2 agonist could reproduce them in a CB2 antagonist-sensitive manner. Importantly, in agreement with previous data [27], we found that RBL-2H3 cells do express CB2, but very little CB1, receptors. We also showed that other direct targets suggested for PEA, i.e., PPARα and GPR55, are not expressed in these cells. Unsurprisingly, this receptor expression profile did not change following short-term stimulation of the cells with either SP or PEA or both.

These findings directed our subsequent experiments, with the aim of investigating the mechanism by which PEA may exert a CB2-dependent effect, since, as confirmed also by our present findings (Fig. 7), this lipid mediator is only very weakly active per se at CB2 receptors. We hypothesized that PEA was acting by elevating the levels of endogenous CB2 agonists, as previously found in vitro, in some cell types, as well as in vivo, in dogs and humans (see above). To date, only the two endocannabinoids, AEA and 2-AG, have been identified as endogenous agonists of CB2 and, of these two compounds, only 2-AG is known to act as a full agonist of this receptor [33], thereby producing inflammation and pain modulatory effects both in vivo, such as for example in ACD [16] and in a model of NI-induced pain [54], and in vitro [55]. Thus, we hypothesized that endogenous 2-AG could play a role in the indirect CB2-mediated mechanism of action of PEA. Accordingly, we measured the levels of AEA and 2-AG in unstimulated RBL-2H3 cells and found that they were increased by ~ 2-fold following PEA treatment, in agreement with our previous data on increased levels of AEA and 2-AG by PEA in other cell types [19, 20].

It is known that the activation of the neurokinin-1 receptor by its agonists, such as SP, induces activation of phospholipase C, with subsequent hydrolysis of phosphoinositides into inositol 1,4,5-triphosphate and diacylglycerol [56], which acts as a biosynthetic precursor of 2-AG [4]. Therefore, we speculated that SP-stimulation of RBL-2H3 cells could also induce an increase of endogenous levels of 2-AG. However, we found that PEA increased by ~ 2-fold the amounts of 2-AG also in SP-stimulated RBL-2H3 cells and that, instead, the amounts of 2-AG did not change following stimulation of RBL-2H3 cells with SP alone. This indicates that the higher levels of 2-AG measured in SP-stimulated RBL-2H3 cells treated with PEA were due only to treatment with PEA, which possibly triggered events down-stream to phospholipase C activation, such as, for example, the stimulation of DAGL-β activity in these cells (which do not express DAGL-α), or the inhibition of 2-AG enzymatic degradation. In addition, we found that AEA concentrations did not change in either RBL-2H3 cells only stimulated with SP or SP-stimulated RBL-2H3 cells treated with PEA.

In support of the hypothesis that elevation of 2-AG biosynthesis was both necessary and sufficient to PEA to exert its effects against SP-induced RBL-2H3 cell degranulation, we showed that an inhibitor of DAGLs prevented both PEA stimulation of 2-AG levels and PEA inhibition of degranulation. Furthermore, we found that, in cell-free systems, PEA, at concentrations similar to those necessary to exert the above effects, activated both constitutive DAGL-β activity in RBL-2H3 cell membranes, and human recombinant DAGL-α overexpressed in membranes from COS-7 cells. The effect of PEA on DAGL-α activity in these cells was confirmed by the finding of the significantly increased amounts of 2-AG produced from the enzymatic hydrolysis of the substrate, 1-oleoyl-2-arachidonoylglycerol, in a cell-free system used in the assay of DAGL-α, as assessed by LC-MS. By converse, PEA, at the same concentrations, did not affect MGL activity. Finally, we found that 2-AG, at a concentration of 1 μM, which is not different from that found here in PEA + SP-stimulated RBL-2H3 cells, was able to mimic the MC down-modulating effects of PEA, and, at a subeffective concentration synergized with a subeffective concentration of PEA at producing these effects. These results suggest that PEA is an endogenous activator of 2-AG biosynthesis via the DAGLs, and in particular of DAGLβ, in RBL-2H3 cells, where 2-AG acts as an intermediate of PEA actions.

Previous studies in different models of MC stimulation had shown that (1) 2-AG decreases the immunological activation of guinea pig MC via CB2 receptors [57]; (2) PEA produces a small, but significant reduction in IgE/antigen-stimulated serotonin release at high concentrations, whereas AEA is without effect and 2-AG exerts the opposite effect [46]; (3) AEA inhibits IgE/antigen-induced degranulation of murine bone marrow-derived MC via CB2 and GPR55 receptor activation [58]; and (4) PEA inhibits phorbol ester-induced nerve growth factor release from the HMC-1 MC line via activation of GPR55 [59]. These studies indicate that PEA, 2-AG and AEA may affect in a different manner and via different mechanisms the stability of MC treated with different stimuli, in contexts different from NI, possibly also depending on the PEA receptor expression profile of the cell model used; profile that, in turn, may be modified by mRNA expression modifying stimuli (such as IgE/antigen and phorbol esters) more than by acute treatment with SP, as shown here.

We also investigated whether or not PEA affects the activity, and not only the levels, of 2-AG at CB2 receptors. Using preparations overexpressing the human recombinant CB2, we found that PEA, at the highest concentration tested (which corresponded with the efficacious in vitro concentration, 10 μM), appeared capable to improve the binding affinity of 2-AG and its efficacy in a functional assay only when the endocannabinoid was incubated at nanomolar and almost inactive concentrations. However, while the effect on binding was probably an artifact due to the slight activity exerted by PEA in this assay, the effect on efficacy was likely not biologically significant in the context of the present study, given the fact that 2-AG concentrations in unstimulated and stimulated RBL-2H3 cells were found here to be in the low μM range.

## Conclusions

**Fig. 11:**
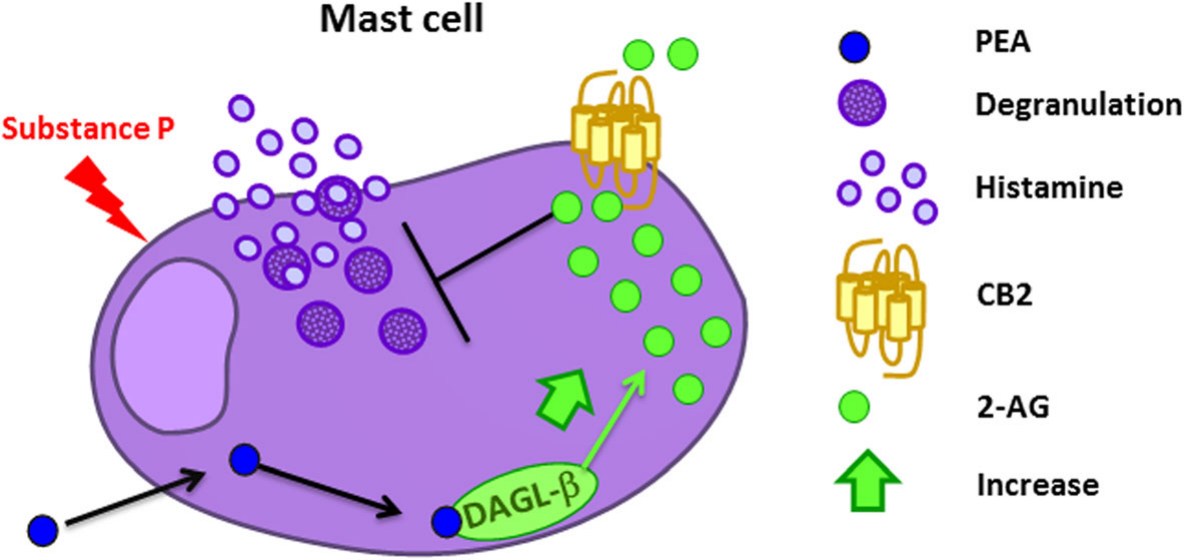
Mechanism of action of PEA in RBL-2H3 cells as suggested by the present study. PEA, by stimulating the activity of DAGL-β, increases the endogenous levels of 2-AG, which, by directly activating the CB2 receptor, blocks histamine release and mast cell degranulation induced by substance P

In summary, we have demonstrated for the first time that short-term treatment with PEA enhances the biosynthesis of 2-AG by stimulating DAGL-α or -β enzyme activity (see the scheme in Fig. 11 for the proposed PEA mechanism of action). Apart from explaining at last the pioneering report of PEA down-regulation of MC hyperactivity [27], this novel mechanism may underlie also the previously described stimulatory effect of orally administered ultra micronized or micronized PEA on 2-AG levels in dogs or humans, respectively [20]. It also suggests that such formulations of PEA may synergize with endogenous 2-AG to modulate NI, as well as other inflammatory processes regulated by the endocannabinoid in blood cells (see [60] for review), via a CB2-mediated mechanism.

## Data Availability

Data generated and analyzed as part of this study are included in the manuscript or are available upon request from the corresponding author.

## Abbreviations

2-AG: 2-Arachidonoylglycerol
AEA: Anandamide
CB2: Cannabinoid receptor type-2
Dagl: Diacylglycerol lipase
Faah: Fatty acid amide hydrolase
MC: Mast cells
Mgl: Monoacylglycerol lipase
Naaa: *N*-acylethanolamine-hydrolyzing acid amidase
Napepld: *N*-acyl phosphatidylethanolamine-specific phospholipase D
NI: Neurogenic inflammation
PEA: Palmitoylethanolamide
PPARα: Peroxisome proliferator-activated nuclear receptor-α
SP: Substance P
TRPV1: Transient receptor potential vanilloid type-1
